# Imaging of Mucosal Inflammation: Current Technological Developments, Clinical Implications, and Future Perspectives

**DOI:** 10.3389/fimmu.2017.01256

**Published:** 2017-10-11

**Authors:** Maximilian J. Waldner, Timo Rath, Sebastian Schürmann, Christian Bojarski, Raja Atreya

**Affiliations:** ^1^Department of Medicine 1, Friedrich-Alexander-Universität Erlangen-Nürnberg, Erlangen, Germany; ^2^Institute of Medical Biotechnology, Friedrich-Alexander-Universität Erlangen-Nürnberg, Erlangen, Germany; ^3^Department of Gastroenterology, Infectiology and Rheumatology, Charité – Universitätsmedizin Berlin, Berlin, Germany

**Keywords:** endoscopy, mucosal inflammation, inflammatory bowel disease, Crohn’s disease, ulcerative colitis, narrow-band imaging, confocal endomicroscopy, multiphoton microscopy

## Abstract

In recent years, various technological developments markedly improved imaging of mucosal inflammation in patients with inflammatory bowel diseases. Although technological developments such as high-definition-, chromo-, and autofluorescence-endoscopy led to a more precise and detailed assessment of mucosal inflammation during wide-field endoscopy, probe-based and stationary confocal laser microscopy enabled *in vivo* real-time microscopic imaging of mucosal surfaces within the gastrointestinal tract. Through the use of fluorochromes with specificity against a defined molecular target combined with endoscopic techniques that allow ultrastructural resolution, molecular imaging enables *in vivo* visualization of single molecules or receptors during endoscopy. Molecular imaging has therefore greatly expanded the clinical utility and applications of modern innovative endoscopy, which include the diagnosis, surveillance, and treatment of disease as well as the prediction of the therapeutic response of individual patients. Furthermore, non-invasive imaging techniques such as computed tomography, magnetic resonance imaging, scintigraphy, and ultrasound provide helpful information as supplement to invasive endoscopic procedures. In this review, we provide an overview on the current status of advanced imaging technologies for the clinical non-invasive and endoscopic evaluation of mucosal inflammation. Furthermore, the value of novel methods such as multiphoton microscopy, optoacoustics, and optical coherence tomography and their possible future implementation into clinical diagnosis and evaluation of mucosal inflammation will be discussed.

## Introduction

Inflammatory bowel diseases (IBDs), which include Crohn’s disease (CD) and ulcerative colitis (UC), affect an estimated 3.1 million people in the United States and about 2.5 million people in Europe. They result in a chronic disabling mucosal inflammation of the gastrointestinal tract (1–3). Affected patients suffer from abdominal pain, diarrhea, hematochezia, weight loss, nausea, etc. and are exposed to an increased risk for complications such as abscess formation, perforation, or the development of colorectal cancer (CRC).

For the clinical diagnosis and management of IBD patients, endoscopic and non-invasive imaging techniques have gained increasing importance for the evaluation of mucosal inflammation during recent years. Although the initial diagnosis of IBD is based on several parameters including clinical, laboratory, endoscopic, radiologic, and histologic features, especially endoscopic results frequently provide essential information for the definitive diagnosis of IBD and the differentiation between CD and UC. Furthermore, endoscopic evaluation of mucosal inflammation vs. mucosal healing is regarded as gold standard for the evaluation of disease activity and therefore the therapeutic management of IBD (3).

Besides inflammation-associated complaints, the increased risk for the development of CRC poses a severe treat for IBD patients. The risk for CRC has been associated with the duration, severity, and extend of colonic inflammation. Independent risk factors include the presence of primary sclerosing cholangitis (PSC) or a family history of CRC. For UC, a cumulative risk of 1.6% after 10 years, 8.3% after 20 years, and up to 18.4% after 30 years has been reported (4). Although recent studies report lower risk rates, for instance Jess et al. described a 2.4-fold increase of CRC risk after 14 years in UC patients (5), it is still widely accepted that long-standing colitis poses a risk factor for CRC development. As a matter of fact, most national and international guidelines on the management of UC recommend repeated endoscopy for CRC surveillance. In patients with CD, an increased risk has been reported in patients with Crohn’s colitis (6). Although data are more limited in comparison to UC, surveillance endoscopy is also recommended for CD patients with long-standing colonic inflammation.

Recent technological developments critically improved the diagnostic accuracy and enabled new applications for endoscopy in various types of diseases and organs. These technologies include wide-field endoscopes with high-definition optical resolution, dye-based or virtual chromoendoscopy, or autofluorescence-endoscopy and also endomicroscopic techniques such as endocytoscopy or confocal laser endomicroscopy (CLE), which provide *in vivo* microscopic information of the mucosal surface during endoscopy. In addition to endoscopic imaging techniques, also non-invasive imaging such as computed tomography (CT), magnetic resonance imaging (MRI), scintigraphy, and ultrasound (US) provide valuable information about disease activity that supplements endoscopic imaging techniques. In this article, we will discuss current data supporting the use of these technologies for the evaluation of mucosal inflammation and provide an outlook on future developments that might further improve the diagnosis and management of IBD.

## Current Endoscopic Techniques for the Diagnosis and Follow-up of Mucosal Inflammation

High-definition video endoscopy is technically mature, widely accepted, and available and therefore considered to be the gold standard for the detection and characterization of mucosal inflammation during initial diagnosis and for evaluating the disease activity in patients with established CD or UC. Whereas conventional white light endoscopy seemed to be sufficient enough for initial and short-term follow-up procedures, more advanced techniques like dye-based and virtual chromoendoscopy or magnification endoscopy are helpful for the evaluation of mucosal healing and in the long-term follow-up during surveillance of IBD.

### Diagnosis and Assessment of Disease Activity

As a first diagnostic step in patients suspicious of IBD, ileo-colonoscopy plays a crucial role for the differentiation of UC and CD. As complementary examinations, upper GI endoscopy, magnetic resonance tomography, small bowel capsule endoscopy (7), or enteroscopy (8) may give additional information on the extent of the disease. In patients with suspected, known or relapsed CD, capsule endoscopy is recommended in those with negative findings in ileo-colonoscopy or gastroscopy (9). The role of colon capsule endoscopy as a surveillance technique, however, is far away from clinical routine and will therefore not replace regular colonoscopies in patients with long-standing IBD in the near future. Except for perianal CD, endosonography of the upper or lower GI tract cannot contribute to the extent of the disease neither for initial diagnosis nor for further evaluation in patients with established diagnosis for IBD. Index-colonoscopy should include a segmental inspection and biopsy of any visible lesion or inflammation in combination with the acquisition of biopsies of non-inflamed mucosal areas (10, 11). The morphological aspect and extent of the inflamed mucosa is of central importance for determining the underlying disease and for distinguishing other inflammatory causes.

**Figure 1:**
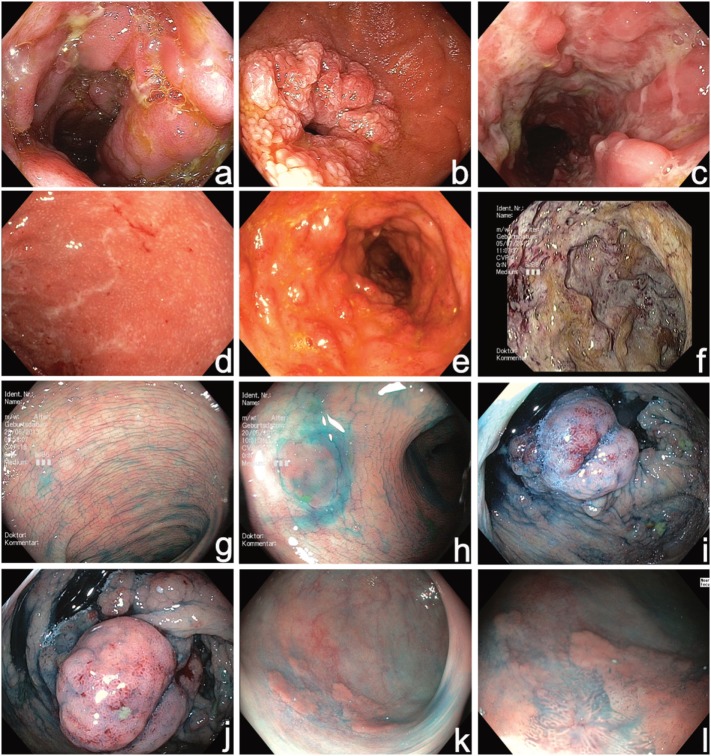
High-resolution video endoscopy used for initial diagnosis of Inflammatory bowel disease (IBD) **(a–f)** and in combination with chromoendoscopy (diluted solution of indigocarmine 0.1%) during surveillance colonoscopy **(g–l)**. **(a)** Acute Crohn’s disease (CD) in the terminal ileum, **(b)** Crohn’s stenosis in the duodenum, **(c)** segmental fissural ulcerations in the left colon SES-CD 32, **(d)** mild active UC UCEIS 3, **(e)** moderate active UC UCEIS 5, **(f)** severe UC UCEIS 8, **(g)** normal chromoendoscopy with uniformly distributed contrast dye, **(h)** identification of a small flat lesion (hyperplastic polyp) with chromoendoscopy, **(i,j)** chromoendoscopy-guided evaluation of pseudopolyps during surveillance colonoscopy, **(k,l)** identification of an inhomogeneous flat polypoid area, and **(l)** with near focus mucosal irregularities are visible indicating high grade intraepithelial neoplasia. SES-CD, simplified endoscopy score for Crohn’s disease; UCEIS, ulcerative colitis endoscopic index of severity.

For an optimal therapeutic management of IBD, regular evaluation of disease activity is mandatory. Endoscopic evaluation of mucosal healing has been shown to provide good correlation with the clinical course of disease and therefore is currently considered as gold standard for evaluating disease activity (Figure 1). In this regard, endoscopic disease activity scores are helpful for the prediction of the disease progression or for evaluation of the treatment success by follow-up procedures after initiation of immunosuppressive therapy. The UC endoscopic index of severity (12), the simplified endoscopy score for CD (13) and the Rutgeerts-Score (14) for the postoperative situation are the most common used scores for documentation of the disease activity in IBD patients. A >50% decrease in Simple Endoscopic Score in Crohn’s Disease (SES-CD) or a Rutgeerts Score i0-i1 is the definition for endoscopic response (15). However, none of these scores has so far been uniformly accepted as standard for endoscopic evaluation of disease activity.

Besides white-light endoscopy, the determination of the disease activity was evaluated in a prospective study with the use of virtual chromoendoscopy with narrow band imaging (NBI) versus white light endoscopy and a special mucosal vascular pattern was noticed with NBI. The vascular pattern showed a good correlation to histology indicating a more precise grading during ongoing endoscopy with NBI (16). Another study found similar results when comparing high-definition white light endoscopy with i-scan virtual chomoendoscopy in patients with IBD (17). In a recent study, optical enhancement with i-scan was combined with magnification endoscopy and a good correlation with histological scores of acute and chronic inflammation was found indicating that this technique might be able to adequately evaluate mucosal healing (18).

However, further data are required to clearly evaluate the usefulness of these techniques for clinical routine endoscopy of IBD diagnosis and monitoring disease activity.

### CRC Surveillance

Although the risk of CRC in IBD nowadays is considered to be lower than previously assumed (19), the overall risk of CRC in IBD patients remains higher in comparison to the general population (20). Therefore, much strength has been made to detect early changes of mucosal alterations in between the active disease periods. Advanced endoscopy techniques, especially dye-based endoscopy, are recommended for the detection of intraepithelial neoplasia (IEN), which has a high risk of progression to IBD-associated CRC. This is not only relevant for high-grade IEN, but also low-grade IEN, which was found to develop infrequently into more advanced neoplasia (21), but has a substantial risk of progression into advanced cancer (22).

For colon cancer screening in the general population, virtual chromoendoscopy (NBI, i-scan, FICE) is not recommended as a standard technique, because comparative studies with high-definition video endoscopy showed controversial results (23–25). No difference was seen when conventional and high-definition white light endoscopy was compared for polyp detection in the general population (26). Classical dye-based pan-chromoendoscopy, mostly used with diluted indigocarmine solution (0.1–0.5%), however, is superior to white light endoscopy and markedly enhanced the detection rate of adenomas in the average risk population (27). Unfortunately, dye-based chromoendoscopy is not used for screening colonoscopy in most Western countries, because this technique is time-consuming, not reimbursed and therefore not well accepted in general practice. Previous studies have also shown a learning curve by using virtual chromoendoscopy techniques for the detection of colorectal neoplasia (23) and further technical improvements like blue laser imaging (28) or linked-color imaging (29) will bring new data regarding CRC screening with virtual chomoendoscopy in the general population.

Although a substantial number (17–28%) of patients with IBD develop CRC before the initiation of a structured surveillance program (30), follow-up colonoscopies are recommended after 8–10 years of extensive colitis or 15–20 years of left-sided colitis (10). A risk stratification should be made for those patients with severe inflammation, colitis-associated PSC, or familiar history of CRC (10). In the surveillance of IBD, dye-based chromoendoscopy with acquisition of targeted biopsies is recommended according to several guidelines (10, 11, 31) and has largely replaced classical random biopsy protocols in most countries. The cost-effectiveness and the efficiency of this surveillance strategy was shown in several studies (32–34). A combination of chromoendoscopy with magnification endoscopy was investigated in very few studies and found a better prediction of disease extent in UC (35). In most Western countries, magnification endoscopy is not used for routine surveillance endoscopy with a more widespread use in the Eastern part of the world. Overall, there is a recognizable tendency toward more sophisticated improvements with the potential to contribute to a better identification and differentiation of colorectal lesions. For instance, first data on full-spectrum endoscopy, a technology providing a field of view of 330°, and the impact on surveillance colonoscopy in IBD patients was published. The authors reported a superior detection rate of cancer precursors and found a large miss rate in forward-viewing endoscopes (36). However, additional studies are still required to fully evaluate the potential of these technologies for IBD surveillance.

Although 10–20 years ago the detection of IEN associated with IBD in many cases directly led to proctocolectomy, published data over the last decade have induced a paradigm-shift toward endoscopic resection techniques, if technically feasible (37). After detection of dysplasia, lesions should be fully resected by endoscopy following the guidelines of the SCENIC consensus conference (38, 39). The development of endoscopic resection techniques and newer data on their safety and efficiency has justified this strategy.

## Endomicroscopy of the Gastrointestinal Tract: *In Vivo* Histology of Inflammatory Diseases

### Principles and Technical Background of CLE

Confocal laser endomicroscopy has been introduced in 2003 and since then, has emerged as a cross-sectional high resolution technique that allows to precisely visualize and characterize gastrointestinal pathology *in vivo* (40–44) at (sub)cellular level (45). Technically, CLE utilizes low-powered blue laser with a wavelength of 488 nm that is directed through a pinhole onto a defined point of the intestinal mucosa. Upon reaching the tissue, an autofluorescence signal is produced which is reflected and refocused on the detection system. Importantly, this reflected light again passes through a pinhole while scattered light from outside the plane of interest is not detected. This results in increased spatial resolution of the images obtained. The region of interest is scanned in both, the horizontal and vertical planes and thereby provides data on signal intensity for each individual point of interest inside the tissue. The fluorescence signal of each point is then converted into a 2D or 3D image using a computer algorithm enabling histologic imaging with 1,000-fold magnification *in vivo* in real time.

Since CLE depends of the fluorescence signal from the tissue, the application of contrast agents either intravenously or topically is required. Among the intravenous contrast agents, fluorescein is most commonly utilized and usually administered immediately before imaging. Optimal image contrast is achieved with 2.5 to 5 mL of fluorescein and images can be obtained within 30 s up to 60 min after injection (46). Administration of fluorescein results in microscopic visualization of the vasculature, the lamina propria, and the intracellular spaces of the tissue while cell nuclei are not stained with fluorescein. Nuclear staining usually requires topical contrast agents such as acriflavine and cresyl violet which can be applied through a spraying catheter (47, 48). However, there is increasing concern over mutagenic potential conferred by topical contrast agents due to their DNA intercaling properties.

To date, two different CLE systems are available and used in clinical routine, both of which are FDA-approved and CE-certified (49) (Table 1): (i) a probe based CLE system which can be used with virtually any existing endoscope with a working channel ≥2.8 mm diameter (pCLE, Cellvizio, Mauna Kea Technologies, Paris, France) and (ii) an endoscope-based CLE which integrated into a high-resolution endoscope (eCLE; Pentax, Tokyo, Japan) (50–52). However, the eCLE system is no longer commercially available. As a common feature, both eCLE and pCLE emit blue laser light with an excitation wavelength of 488 nm and detect the reflected light between 205 and 585 nm. With eCLE images are acquired with a scan rate of 1.6 frames/s and a resolution of 1,024 × 512 pixels, or at 0.8 frames/s with a resolution of 1,024 × 1,024 pixels. With eCLE, laser power and depth of scanning is manually adjustable (depth: 0–250 µm, power: 0–1,000 µW). The acquired images have a confocal image field of view of 475 µm × 475 µm with lateral and axial resolution of 0.7 and 7 µm, respectively.

**Table 1:** Technical characteristics of probe based and endoscope-based CLE devices.

	Endoscope-based CLE	Probe-based CLE			
	eCLE	GastroFlex	GastroFlexUHD	ColoFlex	ColoFlexUHD
Image-plane depth (μm)	0–250	70–130	55–65	70–130	55–65
Lateral resolution (μm)	0.7	3.5	1	3.5	1
Field-of-view (μm)	475 × 475	600 × 600	240 × 240	600 × 600	240 × 240
Frames per second	0.8 –1.6	12	12	12	12
Magnification	1,000-fold	1,000-fold	1,000-fold	1,000-fold	1,000-fold
Required operating channel (mm)		≥2.8	≥2.8	≥2.8	≥2.8
Length (cm)	120 and 180	300	300	400	400

*eCLE, endoscope-based confocal laser endomicroscopy; pCLE, probe-based confocal endomicroscopy; UHD, ultrahigh definition*.

The pCLE system uses stand-alone confocal miniprobes that are compatible with any endoscope with a working channel ≥2.8 mm diameter. Typically, a single probe can be used for 20 different applications and specific probes are available for various organs within the gastrointestinal tract. With pCLE laser power and imaging plane depth are fixed. Depending on the miniprobe utilized, lateral resolution can range from 1 to 3.5 µm and with field of view of 240 to 600 µm. All probes have image scan rates of 12 frames/s with a 30.000 pixels scanning field, thereby enabling real-time videos of the intestinal mucosa.

Based on these technical characteristics, both CLE systems have specific advantages which are for eCLE its higher resolution, the adjustability of the imaging plane depth, and the possibility to simultaneously obtain biopsies for standard histopathology, whereas the pCLE system can be readily used with virtually any endoscope throughout the entire gastrointestinal tract and also allows to obtain videos of the intestinal mucosa in real time.

### CLE for Assessment of Intestinal Inflammation

The technical application CLE as well as the assessment and interpretation of CLE mages for the evaluation of mucosal inflammation can be rapidly learned. Studies have shown that the performance of individual investigators constantly increases over time and leads to a decreased acquisition time and improved diagnostic accuracy after the first three examinations of pCLE (53). In a recent study by Chang et al., the diagnostic accuracy and learning curve for the identification of mucosal barrier function and mucosal integrity was assessed (54). For this purpose, a total of 180 endoscopic CLE images of the terminal ileum were evaluated for increased intestinal permeability (IP) as assessed by cell-junction enhancement, fluorescein leak (FL), and cell dropout (CDO) by experienced and inexperienced analysts as well as pathologists after a 30-min teaching session (54). As shown in this report, the identification of IP requires only a short learning curve after which a high diagnostic accuracy is achieved.

Various studies have demonstrated that increased IP and barrier dysfunction can well be visualized with CLE. As originally described by Kiesslich et al. (55), epithelial gaps are the morphologic equivalent of shedded epithelial cells and these epithelial gaps have been shown to be of utmost importance for the assessment of inflammatory activity with CLE in IBD patients.

**Figure 2:**
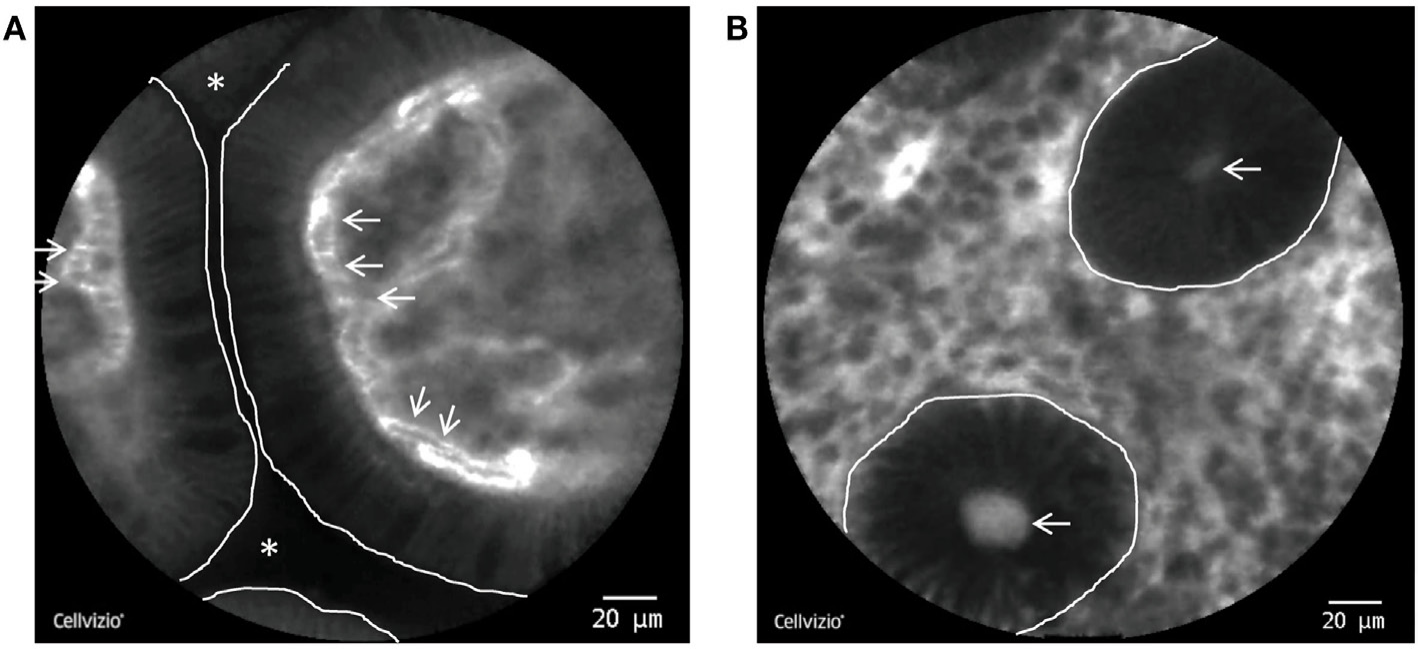
pCLE of the terminal Ileum and the colon. **(A)** Single villi in the terminal ileum as visualized by pCLE. The enterocytes do not exhibit gaps or leakage and the intestinal lumen is not contrasted, consistent with an intact epithelial barrier. White line: border of the enterocytes to the intestinal lumen. White stars: intestinal lumen. White arrows: erythrocytes inside fluorescein containing capillaries. **(B)** Inflamed colonic mucosa from a patient with Crohn’s disease (CD). The dark round structures represent single crypts (white line) with a fluorescein leakage into the lumen (white arrows).

Endomicroscopic characteristics of impaired intestinal mucosal barrier function have been described as the following: (i) FL, in which fluorescein spills into the lumens between two or more shedded or eroded enterocytes, (ii) cell junction enhancement, which, as an apical accumulation of fluorescein between two epithelial cells, morphologically represents an impairment of tight-junction proteins and can therefore be regarded as a precursor of final breakage of the basal tight junction (leading to FL), and (iii) CDO as defined as shedding of apoptotic cells into the luminal space, where they often can be found as cell detritus (Figure 2). Of note, all of the features are functional features and can only be observed with dynamic imaging with CLE. Hence, they do not have a histopathologic equivalent.

In a prospective pilot study in 58 IBD patients in clinical remission, Kiesslich et al. were able to show that increased cell shedding with FL can predict subsequent disease relapse within 12 months after the endomicroscopy (56). Specifically, the sensitivity, specificity and accuracy of an endomicroscopic grading system evaluating cell shedding and local barrier dysfunction (the so-called Watson score) to predict a flare were 62.5, 91.2, and 79%, respectively (56).

Similarly, as shown by Liu et al., the epithelial gap density is significantly higher in patients with CD compared to controls (57) and both UC and CD patients with elevated gap density have been shown to be at significantly higher risk for hospitalization or surgery (58). In a recent study, Lim et al. evaluated CLE images of the duodenum of 35 patients (15 CD, 10 UC, and 10 controls) for the number of epithelial gaps, cell shedding and the degree of FL into the lumen (59). In all patients, the duodenum was macroscopically normal and histopathology showed mild and unspecific duodenitis in 7 out of 15 CD patients while all UC patients had histologically normal duodenal mucosa. Importantly, both UC and CD patients exhibited an increased number of epithelial gaps, epithelial cell shedding, and luminal FL compared to controls, thereby suggesting disease activity otherwise not apparent on conventional endoscopy or histopathology (59). In their totality, these data convincingly illustrate that increased IP and local barrier dysfunction can be visualized by CLE and that the appearance of the later is directly associated with disease outcome.

Confocal laser endomicroscopy also has been proven to be able to precisely assess the degree of mucosal inflammation *in vivo* in real-time in IBD and to discriminate between active disease. As shown for UC, colonic crypts appear small and round with an irregular arrangement in remission upon CLE. In contrast, active disease leads to large, irregularly shaped crypts with a chaotic arrangement and an increased numbers of lamina propria capillaries (60).

When grading inflammatory activity as observed during CLE with a four-grade classification system that combines changes of the crypt and microvascular architecture with FL in patients with UC, Li et al. were able to show that these parameters correlated with histology (61). Interestingly, over 50 percent of patients with endoscopic remission had active disease upon histology. In contrast, remission based on CLE was not associated with active disease on histology. Thus, CLE seems to provide more reliable information on UC activity than white light endoscopy in UC (61). Similar observations can be made in CD: Results from our Erlangen group indicate that active CD is characterized by an increased tortuosity of colonic crypts, enlargment of crypt lumens, increased vascularity, microerosions, and higher lamina propria cell infiltrates on CLE, whereas CD in remission is associated with a higher number of crypts and goblet cells in comparison to controls (62). When these criteria were systematically evaluated using a scoring system [Crohn’s Disease Endomicroscopic Activity Score (CDEAS)], endomicroscopic distinction of patients with quiescent and active disease was possible with a median CDEAS score of 2 in quiescent CD and 5 in active CD (62). In their totality, these data demonstrate that CLE allows to precisely assess the degree of mucosal inflammation in IBD patients.

Apart from that and very consistent with the known histomorphological differences between UC and CE, CLE can also be utilized for the *in vivo* differentiation between these two diseases. Specifically, CD is characterized by significant discontinuation of inflammatory signs such as cryptitis and crypt tortuosity on CLE to UC. UC, in contrast, has been shown to appear with a serious and prevalent crypt distortion, reduced number or density of crypts, and an irregular surface during CLE (44).

Another central field of interest is the detection of dysplasia in IBD and particularly in UC, several studies have investigated the value of CLE during surveillance colonoscopy. In a landmark trial published in 2007 by Kiesslich et al., 161 patients with longstanding UC in clinical remission were randomized to get either conventional white light colonoscopy or chromoendoscopy with endomicroscopy (63). For the detection of dysplasia as the primary outcome, random as well as targeted biopsies were obtained in the WLE group whereas in the endomicroscopy group, circumscribed mucosal lesions were first identified by chromoendoscopy and biopsy specimens were taken only in the presence of *in vivo* mucosal irregularities on CLE (63). Strikingly, by using chromoendoscopy with endomicroscopy, 4.75-fold more neoplasias could be detected than with conventional colonoscopy while at the same time 50% fewer biopsies were required (63).

Soon thereafter, a study on 36 patients with a recent diagnosis of polypoid or non-polypoid lesions showed an overall accuracy (97%) and excellent agreement with histology (kappa value = 0.91) when using CLE to distinguish colitis-associated polypoid lesions from sporadic adenoma. These data suggest that CLE might well be utilized for patient stratification into those suitable for endoluminal resection versus those that would require immediate referral for proctocolectomy (64).

Importantly, the aforementioned studies were performed with eCLE. In a pilot study on 22 UC patients, 48 lesions were compared to 87 random locations with by high-definition WLE, NBI, and pCLE. As demonstrated in a report on 22 UC patient with 48 visible lesions, pCLE is feasable with reasonable diagnostic accuracy for dysplasia surveillance in UC (65).

Although not analyzed systematically, the typical appearance of colitis-associated polypoid lesions has been described as dark cells with mucin depletion, goblet cell and a reduced crypt density, a denticulated irregular epithelial layer, distortion and dilatation of the microvasculature, and increased vascular permeability (45, 66).

At the same time, a recent prospective, cohort study including 61 patients with CD from five centers showed only an incremental increase in the diagnostic accuracy when performing eCLE after chromoendoscopy compared to chromoendoscopy alone while the dysplasia rate was generally low in this study (67).

Overall, these aforementioned studies reliably indicate that CLE allows to assess the microscopic degree of inflammation in patients with IBD and thereby enables real-time *in vivo* histology. Importantly, the microscopic evaluation of mucosal inflammation is a central aspect for the assessment of mucosal healing, which serves as an important prognostic and therapeutic parameter in IBD patients (3). Hence, in order to facilitate and optimize both the medical therapy as well as the dysplasia and cancer surveillance of IBD patients, develop into a widely used diagnostic modality in the near future. Further, cumulating evidence suggesting that the evaluation of the intestinal barrier by CLE can to be used prospectively identify patients that are under risk of experiencing a disease flare and therefore enables a risk-tailored patient care.

## Molecular Imaging of Gut Inflammation and Prediction of Therapeutic Response

As discussed above, the field of gastrointestinal endoscopy has experienced a rapid technological development in recent years, leading to advanced imaging methods that enhance the visibility of mucosal structures and mucosal inflammation. Nevertheless, there is still the unmet clinical need for better visualization of specific mucosal lesions. This necessity is especially evident in the detection of precancerous lesions in cancer surveillance. The sensitivity of the aforementioned endoscopic methods is limited by their reliance to solely detect structural alterations, which can often be minuscule, making them impossible for the detection on the anatomical level. The identification of mucosal lesions could be markedly improved by the visualization and characterization of biological processes that occur at the cellular level, which would add a major new dimension to our current diagnostic possibilities. Imaging of certain biological properties could enable the detection of otherwise not identifiable lesions (68–72).

Endoscopic molecular imaging is based on *in vivo* visualization of disease-specific perturbations at the molecular level. This approach aims to not only broaden our diagnostic capabilities but also provides novel insights into the pathogenesis of various diseases of the digestive tract.

### Requirements for Endoscopic Molecular Imaging

The prerequisite for the successful application of molecular imaging procedures is the identification of molecular targets that represent the answer to the posed clinical question. These targets are often the result of basic science research activities that lead to the successful identification of specific cellular proteins critically involved in the immunopathogenesis of diseases. The epitopes that have so far been targeted in molecular imaging studies include Cathepsin B, epidermal growth factor receptor (EGFR), human epidermal growth factor receptor 2 (HER2), Claudin-1, and tyrosine-protein kinase Met (c-Met) for the enhanced detection of colonic adenoma, and EGFR and vascular endothelial growth factor (VEGF) for CRC. In the stomach, MG7 was identified as a marker for gastric cancer and Periostin for esophageal squamous cell cancer. Furthermore, HER2, certain glycans and cyclophilin A (CypA) were used for better detection of Barrett’s neoplasia in the esophagus (70). These research findings of molecular targets that are specific for diseases build the basis for the translational transfer into preclinical and clinical implementation. Another important requirement are molecular probes that elicit specific interactions with the chosen target structure. The ideal molecular probe would possess high target affinity, rapid binding kinetics, deep tissue penetration, low immunogenicity, safe toxicity profile, *in vivo* stability, low cost, and rapid clearance form non-targeted tissue, which would guarantee maximal specificity for the signal (73). Different probes have so far been used in preclinical or even clinical applications. The most common ones are lectins, peptides, antibodies or affibodies. These dyes are then often labeled by bright fluorescent dyes as optical reporters (68, 74). The most common dyes used in the field of molecular imaging are high-affinity fluorophores like Cyanine 5.5, fluorescein isothiocyanate (FITC), or Alexa Fluor 488 that provide a distinct fluorescence emission spectrum from 422 to 900 nm, which can be detected by dedicated fluorescence endoscopes in real-time. Activatable enzymes represent another highly attractive probe class that has so far only been used in preclinical mouse models. They are optically dormant in the absence of disease and generate a bright fluorescence signal in the presence of proteolytic enzymes that are only overexpressed in neoplastic lesions (75, 76). The probes can be applied systemically, which allows distribution throughout the entire body and deep tissue penetration at the cost of a heightened probability of toxic reactions and the requirement of a lead-time prior to examination. Another alternative is topical administration of the probe *via* a spray catheter into the digestive tissue, which allows application in higher doses to achieve an improved image contrast, while markedly reducing the risk of systemic toxicity. The limitation of this administration route is its restriction to the detection of focal disease only (77).

The most suitable endoscopic system for molecular imaging incorporates a wide-field endoscope that allows to precisely distinguish changes of mucosal architecture and to detect fluorescent molecular probes for further on-site characterization. Several devices have been developed and used in various molecular imaging studies. These include custom fiber optical endoscopes with narrowband filters or blue light sources for excitation (78, 79). Also, CLE has recently become one of the most widely used endoscopic devices for microscopic molecular imaging studies (45). It is currently available as a flexible fiber-optic bundle device that can pass through the instrument channel of the endoscope. It provides real-time images with cellular and even sub cellular resolution *in vivo*. The technique has been described in detail in this manuscript before and uses laser light with a wavelength of 488 nm, which matches the peak absorption of FITC, or 660 nm for excitation. The focus of the laser light is directed to a thin imaging plane inside the tissue. The intensity of the light reflected off a given point, which would be the fluorescent probe in the setting of molecular imaging, is then measured in order to compute a virtual image from these data.

### Preclinical Intestinal Endoscopic Molecular Imaging Studies

The visualization of molecular targets in the colon has been the subject of numerous preclinical studies addressing a variety of clinically relevant problems.

*In vivo* preclinical studies regarding the colon have primarily focused on the detection of neoplastic lesions. Impressive results could be provided by Mitsunaga et al., who topically administered an enzymatically activatable fluorescent probe to detect gamma-glutamyl transpeptidase, which is selectively expressed in colonic neoplasia. Using a modified wide-field fluorescence endoscope, it could be shown that the probe detected most high-grade dysplasias and cancer in mice treated with axoxymethane and dextran (80). This approach of visualizing tumor-specific enzyme activity was also applied in xenograft-bearing mice, in which lysine–lysine cleaving proteases were detected in neoplastic tissue with a high tumor-to-background ratio (81). Another study topically applied a near infrared octapeptide specific for colonic dysplasia in an adenoma mouse model and here again successful identification of the neoplastic lesions was achieved (82).

Other studies used fluorescent labeled antibodies for preclinical detection of malignant tumors. First, a fluorescent antibody targeting EGFR antibody was tested against human xenograft tumors in mice. CLE was able to accurately identify EGFR expression in this experimental setting. Possible application in patients was suggested by topical application of this probe to human colonic specimen *ex vivo*, where differentiation between malignant and non-neoplastic tissue was also proven (83). The same group also applied an Alexa Fluor 488-labeled anti-VEGF antibody in murine tumor xenograft models and surgical human CRC specimen. A handheld confocal instrument allowed successful identification of neoplastic tissue (84). In a subsequent approach, the fluorescent-labeled therapeutic anti-EGFR antibody cetuximab was tested. Prediction of response to monoclonal anti-EGFR antibody treatment with cetuximab was shown in a xenograft model of human CRC cells with high or low expression of EGFR injected into nude mice. The CLE-assessed fluorescence intensity after injection of a labeled cetuximab test dose was able to predict response to subsequent targeted therapy with this monoclonal anti-EGFR antibody (85).

### Clinical Intestinal Endoscopic Molecular Imaging Studies

Endoscopic molecular imaging has already made the transfer into clinical studies and evidence for the feasibility of this approach is continuously growing. A first proof-of-principle study for the detection of intestinal tumors was done with an optical probe built from a monoclonal antibody against carcinoembyonic antigen conjugated with fluorescein. The fluorescent antibody was applied topically during colonoscopy in patients with colorectal polyps or tumors. Patients were examined with a wide-field endoscope with an increased optical range through the use of narrow-band filters. The group was able to identify 19 of 25 tumors and importantly no adverse events or immunological side effects were observed (78). Another pivotal landmark trial using CLE to visualize neoplastic cells was done by topical application of a short peptide sequence isolated from a phage peptide library generated from human adenomas, which was conjugated with fluorescein. The topically applied fluorescein-conjugated peptide showed increased binding to neoplastic cells with a sensitivity and specificity over 80% (86).

A recently published study by Burggraaf et al. elegantly examined the ability of an intravenously injected Cy5-labeled GE-37 peptide to detect dysplastic lesions in 15 patients with high-risk for CRC. The peptide was able to specifically bind to c-Met, which is overexpressed in dysplastic crypts. The examination was done with a modified fiber-optic colonoscope that provided fluorescence images 3 h after injection of the peptide. There was an increased uptake of the probe by colonic polyps. Final analysis demonstrated that all 47 tubular adenomas found, showed increased fluorescence intensity, as did 33/42 hyperplastic lesions and 8/41 of the normal mucosa taken in the study. A total of nine additional adenomas were found by this diagnostic method, which were not found by fiber-optic white light examination. Importantly, there was no systemic side effect visible (87).

Another recently published study was able to impressively detect sessile serrated adenomas (SSAs). SSAs have flat, subtle features and are therefore difficult to detect with conventional colonoscopy. Using phage display, the group of Joshi et al. identified a peptide that preferentially binds to SSAs. Performing *in vivo* fluorescence endoscopy in patients, the authors reported that SSAs had a 2.43-fold increased mean fluorescence intensity compared to healthy colonic mucosa. Fluorescence labeling distinguished SSAs from normal colonic mucosa with 89% sensitivity and 92% specificity. The peptide had no observed toxic effects in the study (88). The same group also demonstrated the ability of a multimodal video colonoscope to collect *in vivo* real-time wide-field images of nonpolypoid colonic adenomas using fluorescently labeled peptides (89).

Apart from the early detection of CRC, molecular imaging procedures were recently used for the prediction of therapeutic efficacy of biological therapies in IBD patients. Reliable prediction of therapeutic response is essential in clinical practice in order to avoid exposure of non-responders to an inefficient biological therapy and the associated potential side effects of this treatment. This would moreover enable the treating physician to directly introduce the patient to the best suited biological therapeutic option, which would enable an improved and time-efficient control of disease for the patient. Recently, a Good Manufacturing Practice (GMP)-conform version of an anti-TNF antibody was topically applied in an investigator initiated trial with 25 CD patients to predict response to subsequent anti-TNF therapy (90). As anti-TNF antibodies appear to induce their anti-inflammatory effect primarily by binding to membrane bound TNF (mTNF) on mucosal target cells (91), the identification of such mTNF-expressing cells in the mucosa was used to identify patients responding to subsequent anti-TNF therapy. The number of mTNF positive cells in the inflamed mucosa was quantified *in vivo* using CLE. In this study, patients with increased numbers of mTNF positive mucosal cells had a superior clinical response at 12 weeks (11/12 patients) compared to patients with lower numbers of mTNF mucosal cells (2/13 patients). Clinical efficacy was sustained in the observed follow-up period of 12 months and was associated with the induction of mucosal healing (90). In the field of antiadhesion molecule therapies, a similar approach was tested *ex vivo* in CD patients. Here, CLE was used in conjunction with a topically applied fluorescein-labeled antiadhesion molecule antibody to visualize mucosal integrin expression *ex vivo* in the mucosal tissue of CD patients to predict response to subsequent anti-adhesion molecule therapy. This approach was again based on the assumption of an association between the expression levels and the response to biological therapy directed against a target molecule. In the study, mucosal biopsies of five CD patients with anti-TNF refractory disease were taken for *ex vivo* molecular imaging with the fluorescent anti-adhesion molecule antibody vedolizumab, to visualize the mucosal expression of its target molecule, the α4β7 integrin. CD patients who responded with sustained clinical and endoscopic remission to vedolizumab therapy showed markedly higher expression of the α4β7 integrin than non-responders (92). These results might open new avenues for personalized medicine in the treatment of CD patients and might serve as a possible model approach for other inflammatory disorders that are treated with biologics.

Although endoscopic molecular imaging procedures are currently at an early stage of development in clinical procedures, the feasibility of this method has been impressively proven by various clinical studies. Possible applications include enhanced detection of neoplastic mucosal lesions, identification of dysplasia in inflamed mucosa, and prediction of therapeutic responses to molecular targeted treatment. The major challenge for further application of these studies is regulatory approval, as fluorescent probes are regarded as new investigational drugs by the authorities and therefore require extensive preclinical efficacy and safety data. Furthermore, facilities that provide GMP-compliant environments are need for the synthesis of the fluorescent probes. Nevertheless, the available exciting data of the first molecular imaging studies clearly emphasize the potential of this method that might have an impressive impact on improved future diagnostic and therapeutic algorithms.

## Non-Invasive Imaging of Intestinal Inflammation

Due to the invasiveness of endoscopy and the associated risk for complications, there is an enduring demand for non-invasive modalities to assess mucosal inflammation. Accordingly, standard imaging techniques including CT, MRI, scintigraphy, and US have not only been used for the detection of stenosis and penetrating lesions such as fistula and abscesses but also for the evaluation of disease activity in IBD patients (93). CT is usually performed as CT enterography with oral and i.v. contrast for the detection of bowel wall pathology and abnormal contrast enhancement (94, 95). Parameters used to assess disease activity include wall thickening, enhancement of the mucosa or intestinal wall, mural stratification, comb sign, and enlargement of regional lymph nodes (95). Similarly, MRI is performed following administration of oral contrast as MR enterography (MRE) and wall thickness, increased contrast uptake, edema, and ulcerations are assessed (96). A quantitative index, the Magnetic Resonance Index of Activity, has been developed that incorporates MRI-based features of disease activity based on logistic regression and shows good correlation with endoscopic disease activity. Scintigraphy is mostly performed with 99mTc-HMPAO or 111In-oxine-labeled white blood cells, which accumulate at sites of active disease (97). Regarding transabdominal US, thickening of the intestinal wall, color doppler-based assessment of vascularization, reduced bowel stratification and peristalsis, or compressibility are used as parameters for the evaluation of disease activity (95). As all of these imaging techniques offer a limited spatial resolution, evaluation of inflammation limited to the mucosa is barely feasible.

In fact, most data from clinical trials are available for the evaluation of CD activity, which can be detected more easily due to transmural inflammation. Regarding the evaluation of upper gastrointestinal tract and small bowel disease activity in CD patients, all techniques have been shown to provide comparable results for the evaluation of terminal ileitis. In comparison to CT, MRI, and scintigraphy, coverage of the entire length of the small bowel is limited with US (97). For the evaluation of Crohn’s colitis, MRI and CT provide high accuracy, although data for CT are limited. The diagnostic accuracy of US in Crohn’s colitis depends on the experience of the investigator and the affected location. It has been reported to be comparable to MRI and CT for the evaluation of the sigmoid/descending colon, whereas accuracy is lowest in the rectum (98).

Overall, data on the evaluation of disease activity in UC with non-invasive imaging techniques are limited. Despite the low spatial resolution, current data are promising for MRE. For instance, Oussalah et al. evaluated disease activity in 96 patients with IBD (UC = 35, CD = 61) using MRI with diffusion-weighted imaging (DWI-MRI) (99). In this study, diagnostic accuracy of DWI-MRI to detect endoscopic inflammation was even superior in patients with UC (sensitivity = 89.47%; specificity = 86.67%; AUROC = 0.920) in comparison to CD (sensitivity = 58.33%; specificity: 84.48%; AUROC = 0.779). Further studies also show valuable results for US, whereas data on CT only show moderate correlation with disease severity (97). Therefore, further studies are urgently required to fully estimate the value of non-invasive imaging in UC.

In addition to traditional tomographic imaging techniques, also new technological developments have recently entered clinical research. A promising technique for the evaluation of disease activity in IBD patients is multispectral optoacoustic tomography (MSOT) (100). MSOT allows a precise localization of specific molecules in tissues up to several centimeters of penetration depth through the photoacoustic effect. The photoacoustic effect describes the observation that light absorbed by molecules is inducing thermoplastic expansion, which can be detected as US waves with very high spatial resolution. By subsequently exciting a tissue with several wavelengths, spectral unmixing techniques can be used to calculate the relative contribution of specific molecules to the overall signal with MSOT. In this way and based on their characteristic absorption, oxygenated, and deoxygenated hemoglobin have been shown to be easily detectable by MSOT. In a recent study, Knieling et al. evaluated the use of non-invasive transabdominal MSOT for the evaluation of CD activity (101). Performing MSOT in 108 patients with active CD and remission, the authors could show that MSOT-based measurements of total hemoglobin in the intestinal wall show excellent correlation with the endoscopic degree of inflammation assessed with the SES-CD. In comparison to US, MSOT was superior regarding differentiation of remission and low-grade disease activity. Although these data are encouraging, further studies are needed to evaluate the full potential of this new technique in IBD patients.

## Future Directions for the Endoscopic Evaluation of Mucosal Inflammation

In recent years, a number of new optical technologies have been evaluated with regard to their diagnostic value in endoscopic assessments of organs and tissues. First, there are various imaging techniques to resolve the morphological structure of tissues and also to gain functional information about cellular processes in some cases. Second, there are a number of spectroscopy techniques that resolve the spectral composition of detected light signals with sensitivity to the molecular composition of the sample. Each of the technologies is based on specific interactions of light with matter: elastic and inelastic scattering, absorption, and fluorescence.

Imaging techniques include optical coherence tomography (OCT), multiphoton microscopy (MPM), coherent anti-stokes Raman scattering (CARS) microscopy, and fluorescence lifetime imaging; the main spectroscopy techniques are Raman spectroscopy and Fourier transform infrared spectroscopy.

Optical coherence tomography can be used to image structural features of tissue. Contrast is based on light absorption and reflection in the tissue at interfaces with refractive index changes. The detection principle is interference of light from a reference path with light reflected from the tissue. Changing the length of the reference path by a moveable mirror tunes the focal position in the sample and encodes the axial position of the signal in the so-called time domain OCT technique. Additional scanning of the light beam in the lateral plane allows for three-dimensional tissue imaging. Alternatively, interference patterns can be detected on a spectrometer and be converted to spatial positions by Fourier transform in spectral domain OCT, which has become the current standard for most commercial systems. Typically, resolutions in the range of 10 µm and imaging depths in the range of 2 mm can be achieved.

First results on the evaluation of gastrointestinal diseases with OCT were provided already 20 years ago (102). In 2004, Shen et al. exposed tissue samples from 48 patients with IBD to OCT *ex vivo* (103). According to this study, OCT enabled the identification of transmural inflammation and thereby allowed the differentiation between CD and UC with excellent correlation to histopathology. This study was subsequently supported by *in vivo* endoscopic OCT data in 40 patients with CD and 30 patients with UC, again proposing that OCT can aid the discrimination of CD and UC based on the detection of transmural inflammation (104). Although these data are encouraging, subsequent confirmation in follow-up studies is missing so far. As there is a continuous research in further improving endoscopic OCT and more advanced devices are under development (105), more data on the use of OCT for the evaluation of mucosal inflammation can be expected.

In MPM, a fluorescent molecule is excited by interacting with two or more photons at the same time. Excitation wavelengths are typically in the near infrared range for fluorescence emission in the visible spectrum. Infrared light is less scattered in the tissue than light of shorter wavelengths, thus larger penetration depth can be achieved for imaging. Multiphoton effects are only observed in the small region of the objective focus, where the energy density is highest. Fluorescence signals can therefore be collected close to the back aperture of the objective without the need for a confocal pinhole that blocks light from out-of-focus planes. This way of detection usually achieves a better signal-to-noise ratio and improved image contrast in tissue imaging, especially in deeper layers of the tissue. Penetration depths are typically limited to 150 or 200 µm in dense tissues like colon mucosa, but may also reach to more than 500 µm in brain imaging. MPM is well suited for label-free imaging of tissue based on autofluorescence [two-photon excited autofluorescence (TPEF)] of endogenous molecules like NADH or FAD, which are present in all cells in different quantities. In addition, the extracellular matrix can be visualized through second harmonic generation (SHG) from collagen-I, which is another specific two-photon effect that can be observed only in a few very regular and non-centrosymmetric filament structures. By combining the detection of TPEF and SHG signals, MPM provides a detailed information of biological tissues without the requirement of labeling.

**Figure 3:**
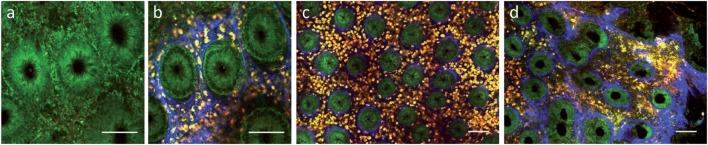
Label-free multiphoton microscopy of Crohn’s disease in human colon biopsies. **(a)** Epithelial layer at 10 µm depth. **(b)** Upper lamina propria at 40 µm depth. **(c)** Weak inflammation. **(d)** Strong active inflammation. Scale bars: 100 µm.

Regarding intestinal tissue, label-free multiphoton imaging can provide a subcellular resolution of the mucosal surface enabling the identification of epithelial cell nuclei, goblet cells, interstitial collagen, etc. (106). Safdarian et al. could show that label-free MPM can be used to detect and quantify eosinophil infiltration in eosinophilic esophagitis *ex vivo* (107). In addition, we have previously shown that label-free MPM can also be used to display mucosal inflammation in tissue samples from IBD patients *ex vivo* (Figure 3) (108). Recent technological progress enabled continuous miniaturization of MPM devices leading to the development of first MPM endoscopy systems, which have been demonstrated in preclinical studies (109, 110). Once clinical MPM devices are available, it will be interesting to see, if this technology can provide further benefit for clinical diagnostics of mucosal inflammation.

Raman spectroscopy is a technique that allows point measurements in biological tissues providing a detailed information about the molecular composition through the detection of inelastic scattering. Inelastic scattering is based on the Raman effect, which can occur due to changes of vibrational, rotational, or electronic energy of a molecule following excitation (111). As individual molecules have characteristic Raman signals, spectroscopic evaluation of these signals provides information about individual molecular components in a tissue sample similar to a molecular “fingerprint.” This information has already been used to the diagnosis of IBD *ex vivo* (112, 113). In a recent study, Addis et al. used Raman spectroscopy to assess disease activity vs. mucosal healing in tissue biopsies of patients with UC *ex vivo* (114). As Raman spectroscopy can be performed with a fiber optic probe, it can easily be integrated into an endoscopy setup. As a proof of principle, Pence et al. used colonoscopy-coupled Raman system in a pilot study in IBD patients *in vivo* (115). The data from this study are encouraging for a future use of Raman spectroscopy in the diagnosis and monitoring of IBD.

In addition to single point measurements, the Raman effect can also be used for tissue imaging through CARS or Stimulated Raman Scattering (SRS) microscopy (116). These technologies probe vibrational molecular transitions, for example C-H bonds. The coherent excitation of these vibrational transitions is achieved by combined excitation with two lasers (called pump and Stokes laser) at two different wavelengths. The energy difference of these two wavelengths is chosen to exactly match the energy of the vibrational bond to be probed in the tissue. The nonlinear interaction of photons from the laser source with molecular oscillators in the sample leads to generation of shorter wavelength photons detected in CARS microscopy and a stimulated Raman loss or gain in scattered light intensities at the original laser wavelengths, which can be probed by lock-in amplifiers in SRS microscopy. In comparison to standard Raman spectroscopy, CARS uses only one Raman frequency for excitation at a time, but with a much higher yield in detected photons that allows for tissue imaging with a laser-scanning microscope. In a recent study, Chernavskaia et al. used label-free non-linear multimodal combining TPEF, SHG, and CARS imaging to evaluate disease activity in tissue samples from IBD patients *ex vivo* (117). Comparing results from these non-linear imaging approaches with histopathological results, the authors could identify a feature set for automatic prediction of disease activity with high diagnostic accuracy. Although these data are preliminary, they propose that non-linear label-free multimodal imaging approaches might be valuable tools for the assessment of mucosal inflammation.

## Conclusion

Endoscopic evaluation of mucosal inflammation has made a significant progress during recent years. New wide-field approaches such as high-definition endoscopy, dye-based chromoendoscopy, or magnifying endoscopy have not only improved the diagnosis and monitoring of disease activity but also cancer surveillance in IBD patients. For the first time, CLE enabled *in vivo* histology of mucosal surfaces providing real-time information about the microscopic state of disease. Despite these improvements, there are still unmet needs for clinical management of IBD patients. First data for molecular imaging approaches or new optical technologies are promising to cover these needs. However, as these techniques are only on the way for clinical translation, future studies will need to show their benefit for the evaluation of mucosal inflammation.

## Author Contributions

MW, TR, SS, CB, and RA together prepared the concept, wrote, and reviewed the manuscript.

## Conflict of Interest Statement

The authors declare that the research was conducted in the absence of any commercial or financial relationships that could be construed as a potential conflict of interest. The handling editor declared a shared affiliation, though no other collaboration, with one of the authors CB.
